# Encephalitis With Antibodies Against the GABA_B_ Receptor: High Mortality and Risk Factors

**DOI:** 10.3389/fneur.2019.01030

**Published:** 2019-09-26

**Authors:** Jingfang Lin, Chen Li, Aiqing Li, Xu Liu, Rui Wang, Chu Chen, Dong Zhou, Zhen Hong

**Affiliations:** ^1^Department of Neurology, West China Hospital, Sichuan University, Chengdu, China; ^2^Department of Neurology, Nuclear Industry 416 Hospital, Chengdu, China; ^3^Department of Pathology, University of Washington School of Medicine, Seattle, WA, United States

**Keywords:** anti-GABA_B_R encephalitis, tumors, prognosis, mortality, predictors of death

## Abstract

**Objective:** To measure mortality, identify predictors of death and investigate causes of death in patients with anti-gamma-aminobutyric-acid B receptor (anti-GABA_B_R) encephalitis.

**Methods:** Prospective analysis of anti-GABA_B_R encephalitis cases diagnosed between June 2013 and August 2018 in West China Hospital of Sichuan University, with assessment of factors associated with mortality.

**Results:** A total of 28 patients (11 females) with anti-GABA_B_R encephalitis were included in this study. After a maximum time of 52 months (median 11 months, range 2–52) of follow-up, 9 (32.1%) patients died, with a median survival time of 6.5 months. Five patients died of tumor progression, one patient died of convulsive status epilepticus, one patient died of septic shock, and two patients died of severe pneumonia. Predictors of death were older age at onset (*P* = 0.025), presence of a tumor (66.7 vs. 15.8%, *P* = 0.013), the number of complications (2.6 vs. 1.0, *P* = 0.009) and deep venous thrombosis (33.3% vs. 0, *P* = 0.026).

**Conclusion:** Patients with GABA_B_R encephalitis have a high mortality rate within 5 years. Older age at onset, presence a tumor, the number of complications, and deep venous thrombosis are associated with death.

## Introduction

Anti-gamma-aminobutyric-acid B receptor (anti-GABA_B_R) encephalitis, which was first described by Lancaster et al. (1), is clinically characterized by limbic encephalitis (including seizures, cognitive disorders, behavioral changes) and other uncommon clinical syndromes (such as cerebellar ataxia and opsoclonus-myoclonus syndrome) (1). Previous studies have reported some patients had additional autoantibodies, such as antibodies targeting voltage-gated calcium channel (VGCC), glutamic acid decarboxylase 65 (GAD65), and sex determining region Y-box 1 (SOX1) (2, 3). Approximately 50% of patients are diagnosed with small cell lung cancer (SCLC), and in rare cases, thymoma, malignant melanoma, breast carcinoma, rectal carcinoma, multiple myeloma, esophageal carcinoma, sarcomatoid carcinoma (SC), and gastric adenocarcinoma have also been found (2, 4–9). The limited number of patients reported since the original series has indicated that patients with anti-GABA_B_R encephalitis have a poor prognosis, particularly in those with tumors (1, 2). However, the prospective studies with respect to long-term prognosis of anti-GABA_B_R encephalitis are lacking and predictors of death are still unknown. Therefore, the primary objective of this prospective study was to assess long-term outcomes of patients with anti-GABA_B_R encephalitis and analyze the possible predictors of death.

## Methods

### Patients

We prospectively enrolled patients with a definitive diagnosis of anti-GABA_B_R encephalitis from the inpatient clinic of the Department of Neurology, West China Hospital between June 2013 and August 2018. The patients included met the following diagnostic criteria for anti-GABA_B_R encephalitis (10): (1) subacute onset (rapid progression of <3 months) of seizures, working memory deficits, or psychiatric symptoms; and (2) positive results for anti-GABA_B_R antibodies in cerebrospinal fluid (CSF) and/or serum. The exclusion criteria were as follows: (1) patients with <3 months' follow-up; (2) patients with laboratory evidence of infectious encephalitis, for example, viral, bacteria, mycobacterium tuberculosis (TB), parasitic, or fungal; (3) patients diagnosed with toxic-metabolic encephalopathy, brain tumor or metastasis, vitamin deficiency or alcohol-related encephalopathy, epilepsy, and/or other nervous system disease prior to the onset of anti-GABA_B_R encephalitis; or (4) patients with positive results of anti-GABA_B_R antibodies in CSF and/or serum but atypical symptoms of encephalitis. Neurologists who had received uniform training on the study protocol interviewed and followed up all of the potential target patients in the inpatient clinic of our center. If the inclusion criteria were met, an experienced neurologist introduced the study to the caregivers and obtained written informed consent from the caregivers or patients prior to enrollment in the study. This study was approved by the Research Ethics Committee of West China Hospital of Sichuan University.

### Clinical Information and Outcome Assessment

Demographics, clinical manifestations, the results of auxiliary examinations and treatment strategies of these patients were collected by an experienced neurologist at the time of diagnosis of anti-GABA_B_R encephalitis. Routine electroencephalogram (EEG) results were assessed by certified neurophysiologists and magnetic resonance imaging (MRI) results were evaluated by experienced neurologists and radiologists. Clinical outcome assessments were collected by follow-up clinic visits from the patient or/and their caregivers. The prognosis of the patients was evaluated by examining the modified Rankin scale (mRS), which was measured every 3 months (11). A mRS score of 0–2 was defined as a good outcome. The cause of death and main complications were evaluated.

### Screening for Antineuronal Antibodies

CSF and serum examinations of patients were performed within 1 week after hospital admission. All specimens were tested for autoimmune or neurologic paraneoplastic antibodies including *N*-methyl-D-aspartate (NMDA) receptors, a-amino-3-hydroxy-5-methyl-4-isoxazol-propionic acid (AMPA) receptors, contactin-associated protein-2 (CASPR2), leucine-rich glioma-inactivated protein-1 (LGI-1), gamma-aminobutyric-acid (GABA) receptors, dipeptidyl-peptidase–like protein 6 (DPPX), lgLON5 or with neurologic paraneoplastic antibodies (anti-Hu, anti-Ri, anti-Yo, anti-CV2, anti-Ma, anti-amphiphysin, anti-Tr, PCA-2, anti-(GAD65) by indirect immunofluorescence assays (IFAs) on human embryonic kidney (293) cells (Euroimmun, Luebeck, Germany). CSF samples with antibody titers of 1:100 or above were defined as strong positive.

### Definition

Status epilepticus (SE) was defined as more than 5 min of (1) a continuous seizure or (2) two or more discrete seizures between which there was incomplete recovery of consciousness (12). Relapse of encephalitis was defined as new onset or worsening of symptoms occurring after an initial improvement or stabilization of at least 2 months (13).

### Statistical Analysis

SPSS version 25.0 (SPSS Inc., Chicago, IL, USA) was applied for the statistical analyses. Fisher's exact test was used to evaluate the differences in the categorical variables. The test for normal distribution was done. Continuous variables with normal distribution were compared using *t* test and continuous variables with non-normal distribution were compared using Mann Whitney *U* test. The survival analysis was performed using the Kaplan–Meier method and the differences were compared using the log-rank test. A two-sided *P* < 0.05 was considered statistically significant.

## Results

### Clinical Characteristics

**Figure 1:**
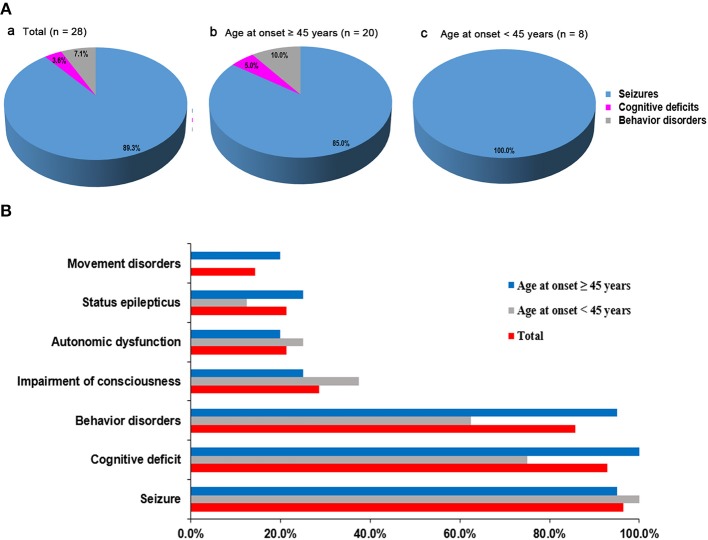
Distribution of initial symptoms and cumulative symptoms during disease course. **(A)** Initial symptom at disease onset according to patients' age. The most common initial symptoms were seizures. In older patients (age at onset ≥ 45 years), 10.0% patients started with behavior changes and 5.0% patients presented with memory deficits as the initial symptom. Seizure as first symptoms occurred in all younger patients (age at onset <45 years). **(B)** Accumulative symptom presentation during disease course in different age groups. No significant statistical differences were found in two different age groups.

We initially evaluated 31 patients with positive results of anti-GABA_B_R antibodies in CSF and/or serum, and three were finally excluded because two patients were lost to follow-up and one patient presented with Isaacs syndrome without encephalitis symptoms such as seizures, working memory deficits, or psychiatric disorders (8). Overall, we included 28 patients who met both the inclusion and exclusion criteria in the study and continued a follow-up with a maximum time of 52 months (median 11 months, range 2–52). Eight of the patients have been published in previous articles (14). Seventeen patients were male (60.7%). Median age at onset of disease was 53 years, ranging from 18 to 75 years. The demographic clinical characteristics and univariable analysis are summarized in Table 1. The most common initial symptoms were seizures (25/28, 89.3%). All of the patients start with seizures were accompanied by typical limbic presentations and the delay from the first initial symptom to the full encephalitis syndrome was 8 days (range 1–30). All younger patients (age at onset <45 years) initially experienced seizures. In older patients (age at onset ≥45 years), two (10.0%) patients initially experienced behavior changes, and one (5.0%) patient presented with memory deficits as the initial symptom (Figure 1A). Accumulative symptom presentation during the disease course in different age groups is shown in Figure 1B. The percentage of patients who progressed to seizures, cognitive disorders, behavior disorders, movement disorders, and decreased consciousness were 96.4, 92.9, 85.7, 14.3, and 28.6%, respectively. Four patients showed movement disorders: two experienced limb involuntary movement; one showed opsoclonus-myoclonus; and one showed gait ataxia. Although no significant differences were found between the two different age groups, older adults had a higher rate of developing convulsive SE (25.0 vs. 12.5%, *p* = 0.640), movement disorders (20.0 vs. 0%, *p* = 0.295), behavior disorders (95.0 vs. 62.5%, *p* = 0.058), and memory deficits (100 vs. 75.0%, *p* = 0.074) during the course of disease than younger adults.

**Table 1:** Characteristics of patients and comparisons between the death group and the survival group.

**Characteristic**	**Total (*n* = 28, %)**	**Death (*n* = 9, %)**	**Survival (*n* = 19, %)**	***p*-value**
Age at onset (years); median (range)	53 (18–75)	60 (51–75)	46 (18–75)	0.025^a^
<45 years	8 (28.6)	0 (0.0)	8 (42.1)	0.029^b^
≥45 years	20 (71.4)	9 (100.0)	11 (57.9)	–
Sex (male)	17 (60.7%)	5 (55.6)	12 (63.2)	1.000^b^
Smoking history	15 (53.6)	5 (55.6)	10 (52.6)	1.000^b^
**Clinical information**				
Seizure	27 (96.4)	9 (100)	18 (94.7)	1.000^b^
Convulsive SE	6 (21.4)	4 (44.4)	2 (10.5)	0.064^b^
Cognitive deficit	26 (92.9)	9 (100)	17 (89.5)	1.000^b^
Behavior disorders	24 (85.7)	9 (100)	15 (78.9)	0.273^b^
Impairment of consciousness	8 (28.6)	3 (66.7)	5 (21.1)	0.035^b^
Autonomic dysfunction	6 (21.4)	2 (22.2)	4 (21.1)	1.000^b^
Movement disorders	4 (14.3)	3 (33.3)	1 (5.3)	0.084^b^
Tumor	9 (32.1)	6 (66.7)	3 (15.8)	0.013^b^
Number of complications (mean, range)	1.5 (0–6)	2.6 (1–6)	1.0 (0–2)	0.009^c^
Relapse	6 (21.4)	3 (33.3)	3 (15.8)	0.352^b^
**Ancillary examination**				
Abnormal routine EEG	18 (75.0)	7 (100)	11 (64.7)	0.130^b^
Abnormal MRI	7 (25.0)	2 (22.2)	5 (26.3)	1.000^b^
CSF cell count >8 cells/uL	17 (60.7)	5 (55.6)	12 (63.2)	1.000^b^
CSF protein >0.45 g/L	9 (32.1)	3 (33.3)	6 (31.6)	1.000^b^
CSF GABA_B_R antibody titers (strongly positive)	7 (25.0)	3 (33.3)	4 (21.1)	0.646^b^
**Treatment**				
Internal to immunotherapy	25 (10–139)	23 (14–139)	25 (10–65)	0.561^b^
MTP + IVIg	11 (39.3)	4 (44.4)	7 (36.8)	1.000^b^
MTP	18 (64.3)	7 (77.8)	11 (57.9)	0.417^b^
IVIg	20 (71.4)	6 (66.7)	14 (73.7)	1.000^b^
Second line immunotherapy	1 (3.6)	1 (11.1)	0 (0.0)	0.321^b^
Without immunotherapy	1 (3.6)	0 (0.0)	1 (5.3)	1.000^b^
**Complications**				
Pneumonia	18 (64.3)	8 (88.9)	10 (52.6)	0.098^b^
Respiratory failure	6 (21.4)	4 (44.4)	2 (10.5)	0.064^b^
Urinary tract infection	4 (14.3)	3 (33.3)	1 (5.3)	0.084^b^
Digestive System	6 (21.4)	3 (33.3)	3 (15.8)	0.352^b^
Deep venous thrombosis	3 (10.7)	3 (33.3)	0 (0.0)	0.026^b^
Admission to ICU	3 (10.7)	1 (11.1)	2 (10.5)	1.000^b^

*SE, status epilepticus; EEG, electroencephalogram; MRI, magnetic resonance imaging; CSF, cerebrospinal fluid; MTP, methylprednisolone; IVIg, IV immunoglobulins; ICU, intensive care unit*.

a *T test was used for comparisons of the continuous variables*.

b *Fisher's exact test was used for numerical variables*.

c *Mann–Whitney U-test was used for comparisons of the continuous variables*.

As for the comorbidities in this cohort, as shown in Table 1, respiratory disorders and subsequent complications were the most common. In particular, pneumonia was present in more than two-thirds of the patients (*n* = 18, 64.3%), followed by respiratory failure, digestive system disease (one upper gastrointestinal bleeding, five liver function damage) and urinary tract infection. Additionally, in the cohort, there were 9 patients with tumors. Among these patients, six had SCLC, one had non-small cell lung cancer (NSCLC), one had pancreatic cancer, and one had lung cancer without confirmed histology. All patients presented with neurologic symptoms that preceded the identification of a tumor. Among them, the tumors in three patients were found ~6 months after onset of disease, but not at the initial screening at the time of disease onset.

All patients had paired serum/CSF samples available. In 25 cases (89.3%), GABA_B_R antibodies were detected in both serum and CSF. In two patients, GABA_B_R antibodies were detected only in the CSF, and in one patient, GABA_B_R antibodies were only detected in the serum. The proportion of patients with a strong positive GABA_B_R antibody titer in the CSF was 25.0% (Supplementary Figure 1A). One patient without a tumor had an additional onconeuronal antibody (anti-Hu: 1:10) in the serum. Routine EEG was available from 24 patients: 12 (50.0%) had diffuse or focal slowing, 6 (25.0%) had epileptiform changes and the others were unremarkable (Supplementary Figure 1B). Brain MRI demonstrated abnormalities in mesial temporal regions on T2-weighted images (T2WI) and fluid-attenuated inversion recovery images (FLAIR) in 5 (17.9%) patients (three bilateral and two unilateral) (Supplementary Figure 1C). The detailed brain MRI results are shown in Supplementary Figure 2.

### Treatment and Follow-Up

Median time from onset of disease to initiation of immunotherapy was 25 (10–139) days. In the cohort, 27 patients received first-line immunotherapy: 18 patients received methylprednisolone (MTP) (1 g/d for 5 days) and 20 patients were treated with intravenous IV immunoglobulin (IVIG) (0.4 g/kg/d for 5 days). Among the patients with tumors, only two patients with SCLC received oncological treatment (chemotherapy and radiation therapy), the seven other patients did not receive any tumor treatment due to patients' will or tumor metastasis. One patient who developed complete loss of short-term memory and a behavior disorder, but no epilepsy, did not receive immunotherapy and spontaneously improved up to mild cognitive impairment in about 1 month.

**Figure 2:**
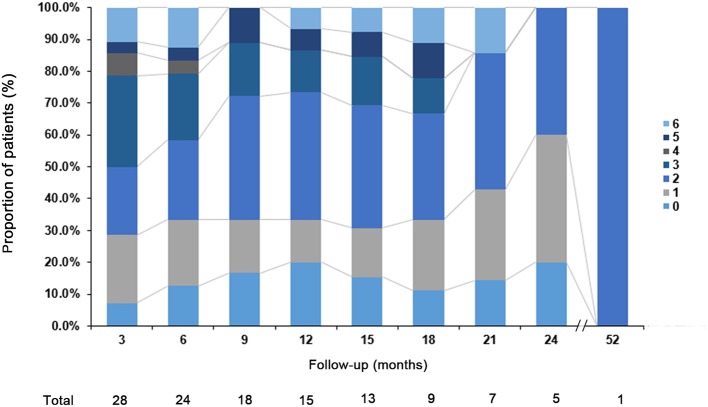
Clinical outcome. The modified Rankin scale (mRS) of the patients with anti-GABA_B_R encephalitis at different follow-up point.

To investigate the prognosis of the disease, the mRS was evaluated over time and these results are summarized in Figure 2. During the first 24 months, 16 of 28 (57.1%) patients had a better outcome (mRS score: 0–2). Three patients (10.7%) were moderately affected (mRS = 3) due to severe cognitive impairment. Two patients who received anti-tumor treatment had partial neurological improvement to immunotherapy with improved mRS (1 and 2, respectively). Most of our patients complained of long-term memory defects and were unable to return to their premorbid baseline status. In addition, our data suggest that the prognosis is worse for older patients (*P* = 0.008). Only two patients still have seizures at 24 months. A total of 21.4% of the patients had clinical relapses and the median time from onset of initial disease to relapse was 6.5 months (range 3–45 months).

### Fatal Cases

During the follow-up, 9 of 28 patients (32.1%) died and the fatal cases are summarized in Table 2. The median age of the patients who died was 60 years, ranging from 51 to 75 years, with five men and four women. The median interval between symptom onset to death was 6.5 (2.5–21) months. Five patients died of tumor progression, which included SCLC, NSCLC, pancreatic cancer, and lung cancer without histology confirmed; one patient from convulsive SE; one patient from septic shock; and two patients with severe pneumonia died soon after discharge from the hospital. All patients in the death group died within 24 months and 7 (77.8%) of 9 patients died within 12 months after disease onset.

**Table 2:** Clinical information of patients who died of anti-GABA_B_R encephalitis.

**No**.	**Sex, age (year)**	**Chief complaint**	**Convulsive SE**	**Tumor**	**Immunotherapy**	**Main complication**	**Interval from onset to death (month)**	**Cause of death**
1^*^	M/59	Repeated GCS for 30 days, behavior disorders for 7 days	Convulsive SE	NSCLC	MTP	Pneumonia	21	Tumor progression
2	M/54	Repeated seizures for 6 days	No	SCLC	MTP	Pneumonia	7	Tumor progression
3	F/60	Headache and fever for 10 days, repeated GCS for 5 days	Convulsive SE	Lung cancer	MTP	Severe pneumonia, respiratory failure, liver damage, venous thrombosis	2.5	Convulsive SE
4	F/56	Headache for 30 days, behavior disorders for 7 days	No	No	MTP+ IVIG	Severe pneumonia, respiratory failure, urinary tract infection	3	Severe pneumonia
5	F/63	Repeated GCS for 3 days	No	Pancreas cancer	IVIG	Severe pneumonia, respiratory failure	6	Tumor progression
6	M/51	Repeated complex partial and generalized seizures for 24 days	Convulsive SE	No	IVIG	Septic shock, severe pneumonia, respiratory failure, gastrointestinal hemorrhage, venous thrombosis, urinary tract infection	3	Septic shock
7^*^	F/62	Psychiatric symptoms for 17 days	No	SCLC	MTP+ IVIG	Urinary tract infection	10.5	Tumor progression
8	M/67	Repeated GTCS for 15 days	Convulsive SE	SCLC	MTP+ IVIG	Pneumonia, liver damage	6.5	Tumor progression
9	M/75	Repeated GTCS for 4 months	No	No	MTP+ IVIG	Severe pneumonia, respiratory failure, Venous thrombosis	20	Severe pneumonia

*GTCS, generalized tonic- clonic seizures; SE, status epilepticus; NSCLC, non-small cell lung cancer; SCLC, small cell lung cancer; MTP, methylprednisolone; IVIG, IV immunoglobulins*.

* *Tumor screening (computed tomography and B-type ultrasonic examination) was initially negative and was only found about 6 months after onset of disease*.

### Comparisons Between the Death Group and Survival Group

Comparative results between the death group and survival group are summarized in Table 1. With regard for baseline clinical characteristics, only the age at onset was significantly associated with mortality (*P* = 0.025). Regarding clinical information, patients in the death group were more likely to have a tumor (66.7 vs. 15.8%, *P* = 0.013). In addition, patients in the death group developed on average close to three (2.6, range: 1–6) kinds of complications, which was significantly higher than that of the survival group (1.0, range: 0–2, *P* = 0.009). Regarding complications, three (33.3%) patients in the death group developed deep venous thrombosis, whereas no patient was diagnosed with deep venous thrombosis in the survival group (*P* = 0.026). Importantly, although there was no significant difference between the two groups (*P* = 0.064), the proportion of patients with convulsive SE and respiratory failure in the death group was higher than that in the survival group. Multivariate analysis could not be applied due to the small number of patients.

**Figure 3:**
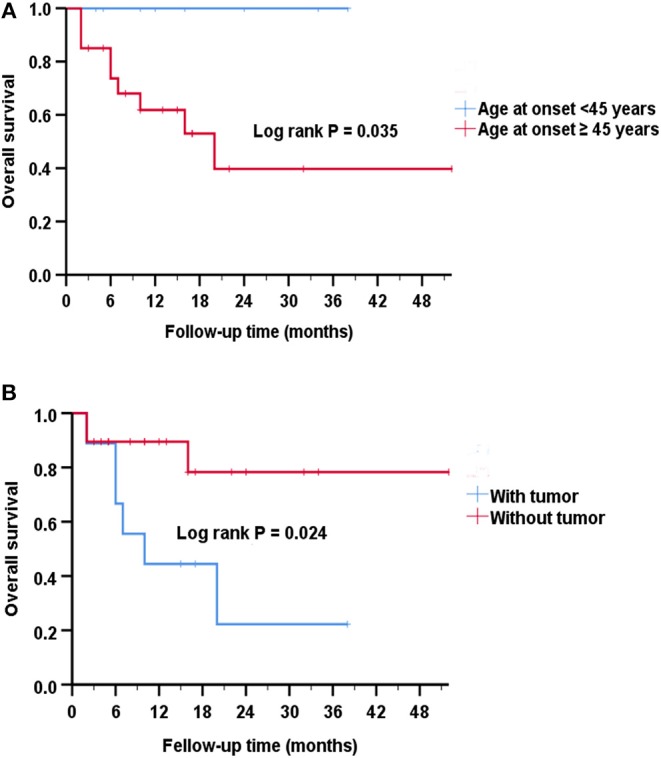
Analysis of Kaplan-Meier survival curve for survival. **(A)** Kaplan-Meier survival curves show that patients with age at onset ≥45 years had an increased risk of death compared to those with age at onset <45 years (Log rank *P* = 0.035). The estimated median survival time was 10 months for the group with age at onset ≥45 years and 14 months for the group with age at onset <45 years. **(B)** Kaplan-Meier survival curves show that patients with tumors had a greater risk of death than patients without tumors (*P* = 0.024). The estimated median survival time was 10 months for the group with tumor and 12 months for the group without tumor.

Overall survival was defined as the period between the date of diagnosis of the disease until the last follow-up or death. By Kaplan-Meier survival estimates, patients with age at onset ≥45 years had a greater risk of death compared to those with age at onset <45 years (log rank *P* = 0.035) (Figure 3A). No significance was observed if using other ages as a cutoff (data not shown). There was a statistically significant association between survival rate and the presence of a tumor (log rank *P* = 0.024) (Figure 3B).

## Discussion

The current study describes the mortality and assessed the predictors of death in Chinese patients with anti-GABA_B_R encephalitis. Three relevant findings are provided: (1) The mortality of anti-GABA_B_R encephalitis in this series was 32.1% (9/28); (2) tumor progression was the most common cause of death in anti-GABA_B_R encephalitis, followed by severe pneumonia and convulsive SE and septic shock; and (3) age, presence of a tumor, number of complications, and deep venous thrombosis were significantly associated with an increased risk of mortality.

This cohort showed that most patients initially have only seizures, and later develop additional symptoms. Such a step-wise progression has been described before by Maureille et al. (15). Compared with previously published studies, we found this cohort had relatively fewer MRI abnormalities in the medial temporal lobe. However, normal MRI results can be observed in anti-GABA_B_R encephalitis, which suggests that even negative MRI results do not exclude the possibility of this disease (1). In addition, it has reported that lower incidence of mesial temporal lobe FLAIR MRI changes of the anti-GABA_B_R encephalitis in China (18.2–57.1%) (14, 16–18), which may result from inadequate brain screening and imaging follow up. Our data showed a much lower rate of stay in intensive care units (ICUs) than in developed countries (10.7 vs. 64%) (15), which may be a result of the high hospitalization costs in the ICU and different baseline characteristics of disease.

In general, the mortality rate shown in our study is lower than Maureille et al. reported in France (41.0%), Lancaster et al. reported in the United States of America (USA) (40.0%) and Hoftberger et al. reported in Spain (40.0%) (1, 2, 15), but it is higher than several other authors who reported in China [22.2% (16), 27.3% (14), 9.1% (17), 14.3% (18)] and Kim et al. who reported in Korea (0%) (19). The differences in mortality found in case series in Europe, the USA and China may be due to low paraneoplastic rate (Europe: 76.2%, vs. the USA: 46.7% vs. China: 36.2%) in China (1, 2, 14–18). The differences may also be due to the small sizes of each series. Compared to other types of autoimmune encephalitis, the 2 years fatality rate of anti-GABA_B_R encephalitis is significantly higher than that previously reported for anti-NMDAR encephalitis (6%) (13) and anti-LGI1 encephalitis (19%) (20), which may be because patients with anti-GABA_B_R encephalitis have a higher risk of a tumor. Interestingly, all patients in the death group died within 24 months of onset.

Our data indicate that patients in the death group were older at disease onset (*P* = 0.025). All patients who died were older than 45 years and the survival analysis showed that patients who were older than 45 years had a proportionally higher risk of death (log rank *P* = 0.035). An older age increased the risk of a tumor and systemic complications, which can cause death. Among 28 people with a definitive diagnosis of anti-GABA_B_R encephalitis, 8 (28.6%) were <45 years old. The proportion of younger adults in this study was relatively higher than that in previous studies, which reported that only ~10 (7.9%) of the 127 patients with anti-GABA_B_R encephalitis were <45 years old (1, 6, 14–18, 21, 22); however, it was lower than a study of cases reported from Spain (35.0%) (2). It has been reported that anti-NMDAR encephalitis is less severe in patients ≥45 years old than in younger adults and the outcome is poorer in older patients (23). Our data showed there is no difference in clinical presentation between the two different age groups, but the prognosis was poorer in older patients, who were affected by severe memory deficits during the follow-up.

The results of this study confirm previous research showing that the main cause of death in anti-GABA_B_R encephalitis is tumor progression (1, 2, 15). Based on our results, patients with tumors were found to have an increased risk of death (66.7 vs. 15.8%, *P* = 0.013). Our data also showed that patients with tumors had a shorter survival compared with patients without tumors (log rank *P* = 0.024), which is consistent with previous research (*P* = 0.029) (2). However, our cohort presented some clinically important differences from patients in previous studies. First, compared to previous studies that reported that ~50% of the patients had an underlying tumor, our data showed only 9 patients (32.1%) were found to have tumors (1, 2), and it also reported only one third of Chinese patients had an underlying tumor (16). The differences may reflect the small series, inadequate tumor screening, and relatively short follow-up for some patients. Second, the patients with tumors in this study (median age: 59.0; range: 42–67) were younger than patients with tumors in a previous study (median age: 67.5; range: 60–77) (2). We also cannot rule out that there are possible differences among different ethnic groups in regards to tumor distribution.

Notably, in our series, three patients were found to have tumors about half a year after discharge, among whom one patient (aged 45 years old) improved significantly after receiving chemotherapy, one (Patient 1) had substantial improvement after immunotherapy but died after a tumor was diagnosed, and one (Patient 7) with metastasis did not receive cancer treatment and eventually died of tumor progression. Therefore, even when a tumor is not detected in the early disease progression stage, repeated tumor screening is extremely important in follow-up. A total of two patients received anti-tumor treatment and still alive. Maureille et al. suggested that early recognition of the tumors and probably more aggressive tumor treatment are important steps toward an improved outcome (15). Further research should aim for larger sample sizes and explore the response of the tumor to chemotherapy or radiation therapy.

When compared to the survival group, the death group was associated with more complications (2.6 vs. 1.0, *P* = 0.009) and deep venous thrombosis (33.3% vs. 0, *P* = 0.023). However, this is not specific for paraneoplastic encephalitis, and merely reflects that these patients frequently have cancers and usually a serious neurological condition, increasing bed confinement and leading to poor general status. Importantly, the major cause of short-term death was primarily related to severe pneumonia. Although the results were of debatable significance due to the small sample size, an intensive supportive therapy, including management of complications such as severe pneumonia and deep venous thrombosis, should be administered to anti-GABA_B_R encephalitis patients, which might greatly decrease the mortality rate.

In this study, no significant difference was found in convulsive SE and respiratory failure between two groups, which have been reported to be associated with the prognosis of encephalitis (24, 25), but because of the limited sample size in this study, the analysis results should be interpreted cautiously and further studies with larger samples are warranted.

Our study has several limitations, foremost being its relatively small sample size due to the rarity of the disease. Further studies are needed to assess the association between appropriate management of the tumor and mortality and conclusively prove risk factors for mortality in anti-GABA_B_R encephalitis.

## Conclusion

Tumor progression was the most common cause of death in anti-GABA_B_R encephalitis. Older age, presence of a tumor, number of complications, and deep venous thrombosis are the main predicators of death.

## Data Availability Statement

The raw data supporting the conclusions of this manuscript will be made available by the authors, without undue reservation, to any qualified researcher.

## Ethics Statement

Research Ethics Committee of the Medical School of Sichuan University has approved the methods and the use of human subjects for this study. We have received the consent from all of our patients or their guardians participating in the study by the form of written informed consent and have them on file in case they are requested by the editor.

## Author Contributions

JL and CL collected data and drafted the manuscript. AL, XL, RW, and CC carried out the statistical analysis and interpreted the data. DZ revised the manuscript. ZH conceptualized and designed the study and revised the manuscript.

### Conflict of Interest

The authors declare that the research was conducted in the absence of any commercial or financial relationships that could be construed as a potential conflict of interest.

## Supplementary Material

Supplementary Figure 1 Auxiliary examination results of patients. **(A)** Antibody titers in CSF. **(B)** Electroencephalogram results are available in 24 patients (normal: 25.0%; diffuse or focal slowing: 50.0%; epileptiform changes: 25.0%). **(C)** Magnetic resonance imaging (MRI) results are available in 28 patients (normal: 75.0%; mesial temporal lobe lesions: 17.9%; leukoencephalopathy: 7.1%).

Supplementary Figure 2 Detailed MRI findings of patients. Brain MRI was performed using a Germany Siemens-Trio Erlangen 3.0 T MRI (12-channel coil). Regular MRI series including axial T2-weighted image (T2WI), T1-weighted image (T1WI), and fluid-attenuated inversion recovery image (Flair). Contrast-enhanced studies using intravenous gadopentetate dimeglumine. Yellow arrow indicated the imaging lesions. **(A)** Hypersignals were seen in bilateral medial temporal lobe (Flair); **(B)** Leukoencephalopathy (T2); **(C)** Pancreatic mass (Abdominal enhanced computed tomography scanning).
