# Supplementary figures and images for: Encephalitis With Antibodies Against the GABAB Receptor: High Mortality and Risk Factors
Source: Front Neurol. 2019 Sep 26;10:1030. doi: 10.3389/fneur.2019.01030 (PMC6798040; doi:10.3389/fneur.2019.01030)

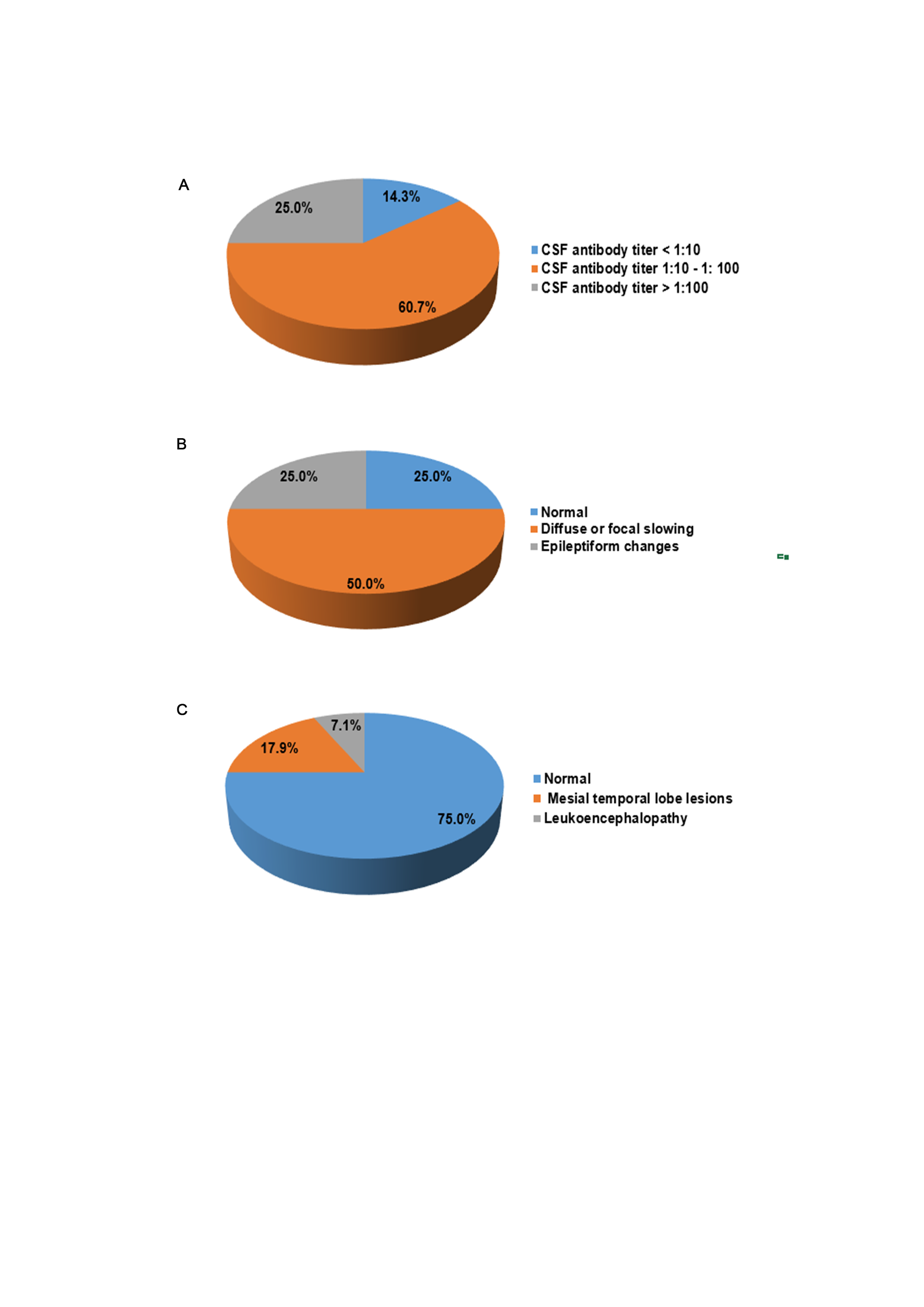

Supplement: Supplementary Figure 1 — Auxiliary examination results of patients. (A) Antibody titers in CSF. (B) Electroencephalogram results are available in 24 patients (normal: 25.0%; diffuse or focal slowing: 50.0%; epileptiform changes: 25.0%). (C) Magnetic resonance imaging (MRI) results are available in 28 patients (normal: 75.0%; mesial temporal lobe lesions: 17.9%; leukoencephalopathy: 7.1%). [file Image_1.tif]

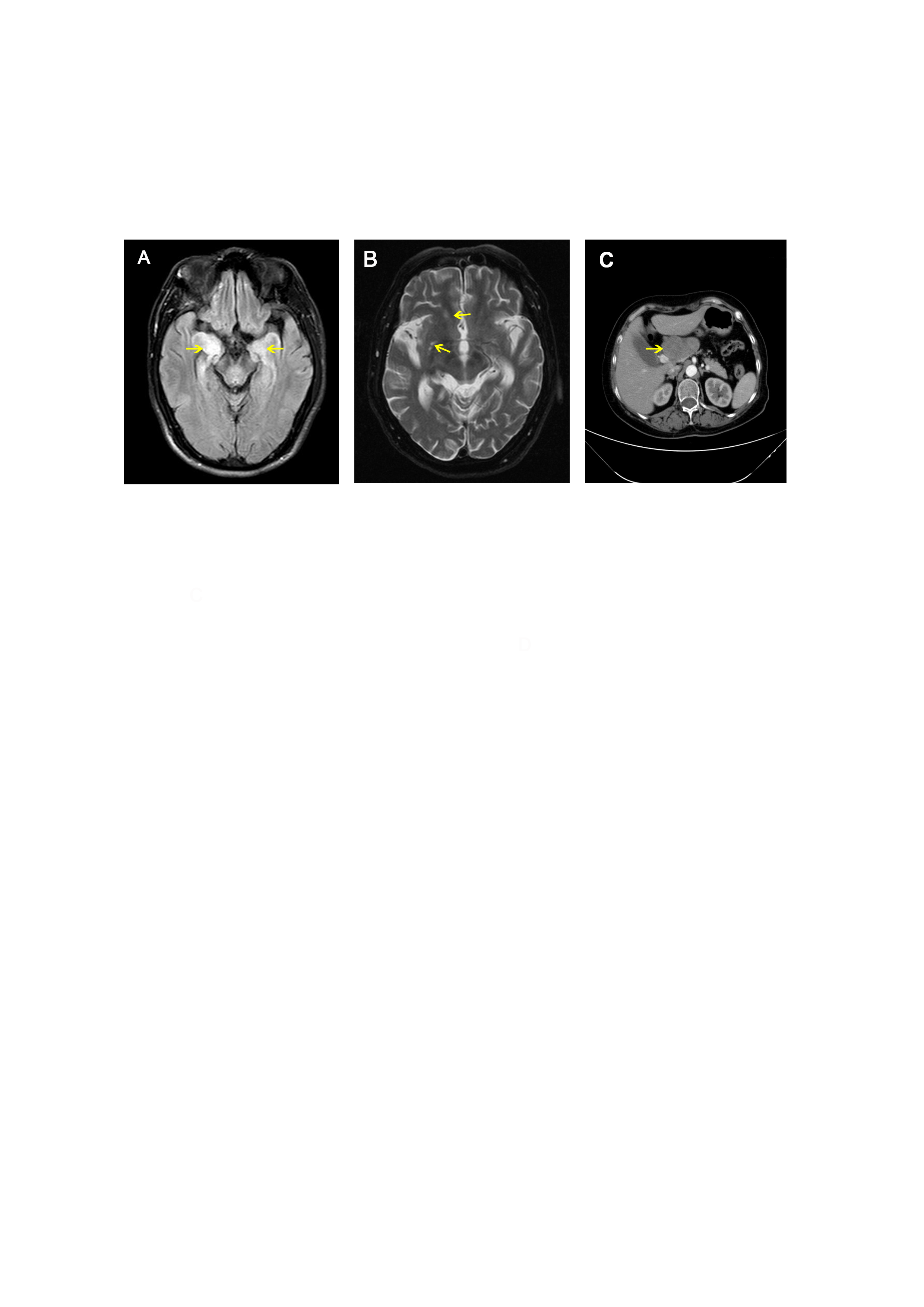

Supplement: Supplementary Figure 2 — Detailed MRI findings of patients. Brain MRI was performed using a Germany Siemens-Trio Erlangen 3.0 T MRI (12-channel coil). Regular MRI series including axial T2-weighted image (T2WI), T1-weighted image (T1WI), and fluid-attenuated inversion recovery image (Flair). Contrast-enhanced studies using intravenous gadopentetate dimeglumine. Yellow arrow indicated the imaging lesions. (A) Hypersignals were seen in bilateral medial temporal lobe (Flair); (B) Leukoencephalopathy (T2); (C) Pancreatic mass (Abdominal enhanced computed tomography scanning). [file Image_2.tif]
